# Effectiveness and safety of electroacupuncture for insomnia: A protocol for an overview of systematic reviews and meta-analysis

**DOI:** 10.1097/MD.0000000000022502

**Published:** 2020-10-02

**Authors:** Xingchen Zhou, Zhenhai Chi, Jun Xiong, Guomin Huang, Ziru Li, Yanan Yang, ShuiSheng Zhou, Rui Yang, Qiangjian Mao, Desheng Wu, Yang Shen

**Affiliations:** a The Affiliated Hospital of Jiangxi University of Traditional Chinese Medicine; b Jiangxi University of Traditional Chinese Medicine, Nanchang, China.

**Keywords:** electroacupuncture, insomnia, overview, protocol

## Abstract

**Background:**

Insomnia is a common disease characterized by difficulty falling and/or staying asleep, and accompanied by irritability or fatigue during wakefulness. It is widely reported that insomnia is one of the most extensive mental disorders which the incidence rate is estimated to be about 10%. Insomnia can have serious influences on patients health and quality of life. Electro acupuncture (EA) is reported to be efficacious and widely used for the treatment of insomnia in China. This overview aims to summarize the available evidence from current systematic reviews for the efficacy of electroacupuncture therapy for insomnia.

**Methods:**

We will make a comprehensive retrieval in 7 databases as following:

1. Embase;
2. Cochrane Library;
3. Pubmed;
4. Chinese databases SinoMed (previously called the Chinese Biomedical Database);
5. Chinese National Knowledge Infrastructure (CNKI);
6. Chinese Scientific Journals Database (VIP);
7. Wanfang Data (WF).

The time is limited from the construction of the library to August 2020. We will use the Assessment of Multiple Systematic Reviews-2 (AMSTAR-2) tool to evaluate methodological quality. Preferred Reporting Items for Systematic Reviews and Meta-analysis Protocols (PRISMA-P) will be used in the report checklist to assess the quality of reports in the study. The Grading of the Classification of Recommendations, Evaluation, Development and Evaluation (GRADE) will be used to evaluate the included SRs and meta-analysis. Our reviewers will conduct systematic reviews, qualification evaluation, data extraction, methodological quality and evidence quality screening in pairs. The outcomes of interest include: The Pittsburgh Sleep Quality Index (PSQI), The Insomnia Severity Index (ISI), Athens Insomnia Scale, Sleep parameters measured by either subjective or objective approaches, such as actigraphy, polysomnogram, and electroencephalogram. Or any other scale used to assess the level of illness. The evidence will be synthesized where appropriate based on patient subgroups and outcomes.

**Results:**

The results will be published in a peer-reviewed journal.

**Trial registration number:**

INPLASY202080087.

**Conclusion:**

This overview will provide comprehensive evidence of EA for patients with insomnia.

## 1 Introduction

Insomnia is a high incidence of adults, and its therapeutic effects are often unsatisfactory.[^1^,^2^] The main clinical manifestations are difficulty falling asleep, low sleep quality, easy awakening, forgetfulness, and daytime sleepiness.^[3]^ According to the survey, about 12% to 20% of people can be diagnosed with insomnia, and 35% to 50% of adults have related symptoms.[^4^,^5^] Recent studies have shown that insomnia can also lead to cardiovascular and cerebrovascular diseases, endocrine disorders, and mental illnesses.[^6^,^7^,^8^] Not only that, insomnia, and cause apparent economic losses to society.^[9]^ Therefore, it is essential to research the treatment of insomnia better to relieve patients physical and mental health.

Modern medicine believes that old age, women, internal medicine, and mental illness can all cause insomnia.^[10]^ Studies have shown that older adults with respiratory symptoms and physical disabilities increase insomnia risk.^[11]^ Not only that, but taking some drugs can also cause insomnia, including beta-blockers, glucocorticoids, nonsteroidal anti-inflammatory drugs, decongestants, and antiandrogens.^[12]^ The etiology and pathophysiology of insomnia are also inseparable from heredity and the environment.^[13]^

Western medicine treatment of insomnia is mainly divided into psychotherapy and drug treatment. The most commonly used methods of psychotherapy mainly include brief insomnia behavior therapy and cognitive insomnia behavior therapy. They can effectively improve sleep and have almost no side effects.[^14^,^15^] However, its clinical practice is limited by the lack of well-trained therapists and poor patient compliance.[^16^,^17^] Clinical drugs mainly include benzodiazepines, non-benzodiazepines, melatonin receptor agonists, orexin receptor antagonists, and antidepressants with hypnotic effects.^[18]^ However, the dependence of drugs, the possibility of abuse, withdrawal syndrome, and the adverse effects of these drugs’ long-term use also bother doctors and patients.^[19]^ These risk factors prompt patients to seek safer and more effective alternative therapies, such as EA, which is considered an excellent choice by the general public.

Traditional Chinese medicine(TCM) believes that the central disease of insomnia lies in the heart, closely related to the dysfunction of the liver, spleen, and kidneys.^[20]^ Based on TCM's theory, its basic pathogenesis is the loss of support or disturbance of mind.^[21]^ And the imbalance between “yin” and “yang” is considered to be the total pathogenesis of insomnia.^[22]^ Not only that, the meridian theory of TCM believes that the dysfunction of Yinqiao meridian and Yangqiao meridian is also a significant cause of insomnia.^[23]^

EA is one of the most popular TCM techniques used routinely in Asian countries and has a long history of treating insomnia. Many literature reports believe that EA has a unique clinical effect on insomnia.[^24^,^25^,^26^] Studies have found that EA can alleviate the abnormal excitement of the sympathetic nerve epithelial medulla system of insomnia rats, thereby effectively treating insomnia.^[27]^ Not only that, EA can also regulate the balance of neurotransmitters in the brain, such as GABA and Glu, the 2 most crucial amino acid neurotransmitters involved in sleep regulation.^[28]^ In addition, EA can also reduce the levels of IL-1β, IL-2, and TNF-α in the hippocampus of the brain, thereby improving sleep quality.^[29]^

Many randomized controlled trials (RCTs) have confirmed the efficacy of EA in insomnia treatment.[^30^,^31^,^32^] Many meta-analyses also show that EA treatment has specific benefits for insomnia patients.[^33^,^34^] However, there has not been a rigorously designed overview to evaluate the systematic evaluation of insomnia by EA. Therefore, this study evaluates and summarizes the clinical research literature of EA in the treatment of insomnia at home and abroad to provide evidence-based medicine for clinical practice.

## 2 Methods

### 2.1 Study registration

This protocol was designed in accordance with the methodological guidelines for overviews provided by the Cochrane Handbook for Systematic Reviews of Interventions.^[35]^ It is registered on the International Prospective Register of Systematic Reviews. (Registration number INPLASY202080087; https://inplasy.com/inplasy-2020–8-0087/.)

### 2.2 Inclusion and exclusion criteria

PICOS will be applied, including Population, Intervention, Comparison, Outcome, and Study.

#### 2.2.1 Type of study

It only includes systematic reviews and meta-analysis of randomized controlled trials (RCT) on EA in insomnia patients published in English and Chinese.

#### 2.2.2 Type of participants

It will include a systematic review of people diagnosed with insomnia. Regardless of gender, race, occupation, education, nationality, etiology, and severity, all insomnia study participants of all ages can be included.

#### 2.2.3 Type of interventions

One therapeutic intervention used in the experimental group is that EA will be included.

#### 2.2.4 Type of comparator (s)/control

The control group can be a blank control group, a placebo group, a psychological control group or a drug treatment group. If the combination therapy has the same 2 groups, it will also include the combination of EA and other therapies.

#### 2.2.5 Types of outcome measurements

##### 2.2.5.1 Primary outcomes

The Pittsburgh Sleep Quality Index (PSQI) is widely used to assess a persons sleep quality. It consists of 19 self-evaluation items and 5 other evaluation items. The score will indicate the level of sleep quality and the severity of the sleep disorder.^[36]^

##### 2.2.5.2 Secondary outcomes

Secondary outcomes mainly include the following aspects:

1. The Insomnia Severity Index (ISI).
2. Athens Insomnia Scale.
3. Sleep parameters measured by either subjective or objective approaches, such as actigraphy, polysomnogram, and electroencephalogram.
4. Adverse effect, such as vomiting, nausea, or dizziness.

#### 2.2.6 Study Design

SRs containing more than 1 RCT were included. There is no systematic reviews, no separate summary of RCT data, and abstracts without sufficient data will be excluded.

### 2.3 Search methods for identification of studies

We searched three foreign electronic databases (Cochrane Library, Embase, Pubmed) and 4 Chinese electronic databases (China National Knowledge Infrastructure (CNKI), WangFang Database, Chinese Biomedical Literature Database (CBM) and Chinese Scientific Journal Database (VIP) to collect potential systematic reviews (SRs) from their inceptions to August 2020. The language of publication is limited to Chinese or English. The following search terms will be used:Disorders of Initiating and Maintaining Sleep, Disorders of Initiating and Maintaining Sleep, Early Awakening, Nonorganic Insomnia, Insomnia, Primary Insomnia, Transient Insomnia, Rebound Insomnia, Secondary Insomnia, Sleep Initiation Dysfunction, Sleeplessness, Insomnia Disorder, Psychophysiological Insomnia, Electroacupuncture, Electroacupuncture, complementary therapy of electroacupuncture, Traditional Chinese therapy of electroacupuncture, systematic review, meta-analysis, et al. A draft search strategy using Pubmed, one of the planned electronic databases to be searched, is presented in Table 1.

**Table 1:**
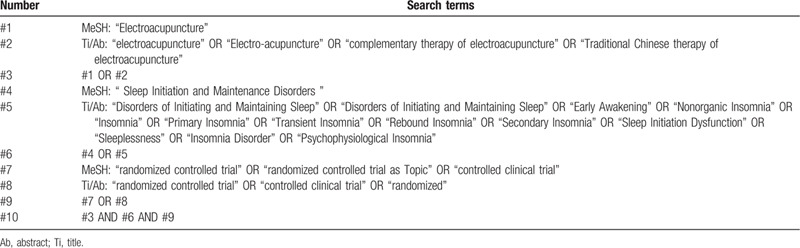
The search strategy for Pubmed.

### 2.4 Studies selection

**Figure 1:**
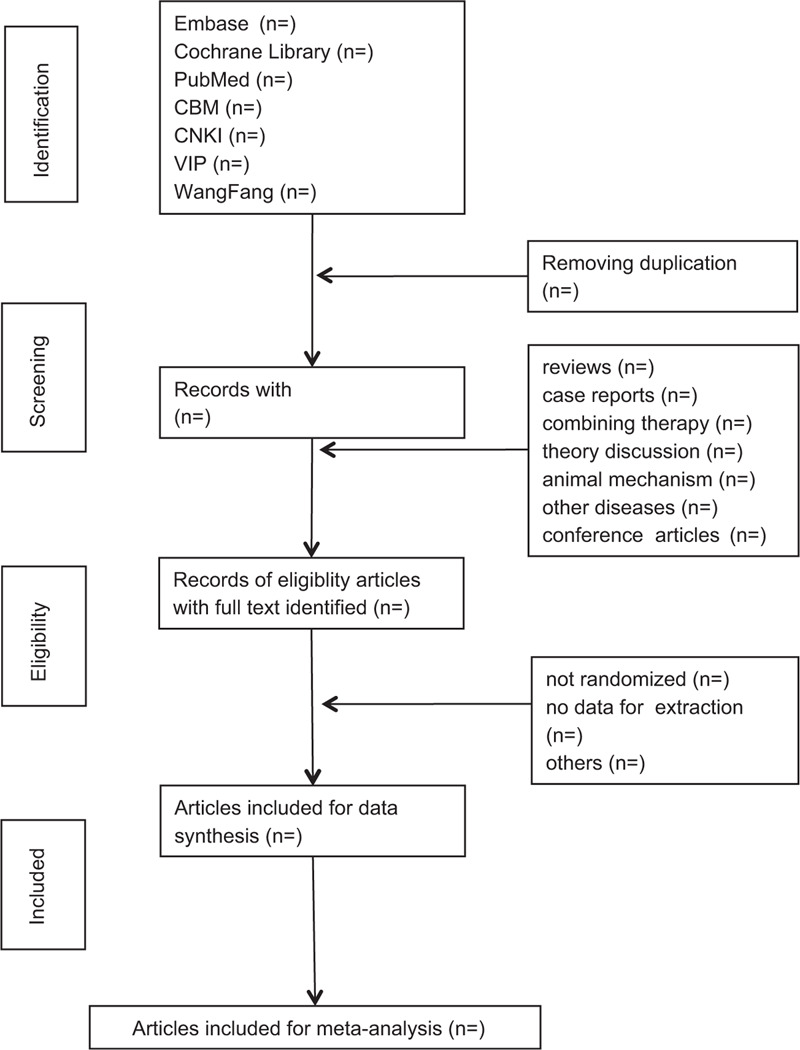
Flowchart of literature selection.

Studies will be identified using NoteExpress 3.2.0. After the initial removal of duplicate studies, 2 reviewers (ZHC and ZRL) will independently screen titles and abstracts based on the eligibility criteria. Full-text studies will be retrieved for all potentially includable SRs or SR protocols. If studies contain insufficient information to make a decision about eligibility, GMH will try to contact authors of the original reports to obtain further details. During the procedure, disagreements will be resolved by discussion or consensus with the third reviewer (RY). Study selection will be performed in accordance with the PRISMA flowchart (Fig. 1).

### 2.5 Data extraction

Two researchers (YNY and SSZ) extracted literature information based on inclusion and exclusion criteria, including the following:

1. Study characteristics: author, year, study design, sample size and follow-up time;
2. Patient characteristics: age, sex, and type of SP;
3. Intervention: intervention measures in the experimental group, intervention measures in the control group);
4. Outcome of the study: Two researchers (DSW and QJM) cross-checked the extraction results of the extracted documents. If there is any difference, you should consult a third party (ZHC) to resolve.

### 2.6 Evaluate the methodological quality of included studies

Two reviewers (XCZ and ZHC) will use the Assessment of Multiple Systematic Reviews 2 (AMSTAR-2) measurement tool to independently assess each SRs methodological quality that meets the inclusion criteria.^[37]^ This is most commonly used to assess the quality of systematic reviews included in overviews. AMSTAR-2 is an update of AMSTAR, which can be used to appraise SRs of both randomized and non-randomized controlled trials. AMSTAR-2 includes 16 items, with each of the 16 criteria given a rating of “yes” (definitely done), “no” (definitely not done), “cannot report” (unclear if completed), or “not applicable” based on the information provided by the systematic review, when the standards are met, the evaluator will evaluate the evaluation. Disagreements will be resolved through discussions between them and arbitrated by the third general author (YS) if necessary.

### 2.7 Evaluation of the reporting quality of the included studies

The 2 authors of the overview (ZHC and JX) will independently evaluate the reports’ quality in each review to assess whether they meet the criteria specified in Preferred Reporting Items for Systematic Reviews and Meta-analysis Protocols (PRISMA-P).^[38]^ If there are any differences, they will be resolved through discussion between them and arbitrated by the third general author (XCZ) if necessary.

### 2.8 Evaluation of the evidence quality of the included studies

The quality of evidence of the included SRs was assessed by the Grading of Recommendations Assessment, Development and Evaluation (GRADE) approach.^[39]^ This tool aims to assess the quality of evidence for each outcome indicator in the study. The 2 authors (ZRL and GMH) will independently evaluate the evidence of the results and should describe in detail the degradation or upgrade factors that affect the quality of the evidence to ensure the reliability and transparency of the results. Any disagreements will be resolved through discussion by 2 authors. The overall quality of evidence was judged as”high,” “moderate,” “low,” or “very low.”

### 2.9 Dealing with lost data

If there is no specific data or insufficient data in the published SRs, the author will be contacted by email or phone to provide the necessary information. If we cannot obtain enough data, the data will be discarded. The analysis will be based on the available data and the potential impact of missing data will be discussed.

### 2.10 Synthesis of data

Before data synthesis, the included SRs and meta-analysis should be considered. For different situations, different measures will be taken for overlapping basic research: whether the basic research completely overlaps, the comment with the highest quality will be selected. If the main research partially overlaps, when the lower quality reviews include more than one-third of the new research. If the basic research does not overlap, the 2 comments will remain. The quality of the review will be fully assessed Use ROBIS and AMSTAR-2. Besides, RevMan5.3.5 software will be used to calculate the standardized effect. The random-effects model (*I* ^2^ ≥ 50%) or fixed-effects model (*I* ^2^ < 50%) will be selected according to the heterogeneity levels of the included SRs and meta-analyses. If the *I* ^2^ value is higher than 75%, the clinical or methodological heterogeneity will be explored through discussion with the review team. When the meta-analysis is not possible, a narrative analysis will be performed. Indirect comparisons of different EA therapies will also be conducted using relative effectiveness outcomes including relative sensitivity and relative specificity.

## 3 Discussion

Insomnia is a disease that frequently occurs at any age. It not only seriously affects the work and life of patients, but also produces depression and anxiety. EA as an effective technique of TCM, has been accepted for insomnia in China. However, due to the lack of rigorous review evidence for EA treatment, clinicians cannot choose the best method. As a result, patients with insomnia are prone to delays. Therefore, the results of this overview will provide real and reliable research evidence for the treatment of insomnia with EA.

The study also has some defects as follows: low quality of original researches, the possible occurrence of false positive or false negative results, various duration of disease different dosage, and frequency of intervention, language restriction, etc. All of these will lead to some bias and influence the results of evaluation results, ultimately affecting this studys reliability.

## Author contributions

**Conceptualization:** Xingchen Zhou, Zhenhai Chi.

**Data curation:** Xingchen Zhou, Jun Xiong, Guomin Huang, Ziru Li, Yanan Yang, Rui Yang, Shuisheng Zhou, Qiangjian Mao, Desheng Wu, Yang Shen.

**Formal analysis:** Xingchen Zhou, Jun Xiong.

**Investigation:** Xingchen Zhou, Zhenhai Chi, Jun Xiong.

**Methodology:** Xingchen Zhou, Guomin Huang, Ziru Li, Yanan Yang.

**Software:** Jun Xiong, Shuisheng Zhou.

**Supervision:** Zhenhai Chi, Jun Xiong.

**Writing – original draft:** Xingchen Zhou, Zhenhai Chi, Rui Yang, Qiangjian Mao.

**Writing – review & editing:** Zhenhai Chi, Jun Xiong, Desheng Wu, Yang Shen.
